# IoT Device Integration and Payment via an Autonomic Blockchain-Based Service for IoT Device Sharing

**DOI:** 10.3390/s22041344

**Published:** 2022-02-10

**Authors:** Anas Dawod, Dimitrios Georgakopoulos, Prem Prakash Jayaraman, Ampalavanapillai Nirmalathas, Udaya Parampalli

**Affiliations:** 1 Department of Electrical and Electronic Engineering, Melbourne School of Engineering, The University of Melbourne, Melbourne, VIC 3010, Australia; nirmalat@unimelb.edu.au; 2 School of Computing and Information Systems, Melbourne School of Engineering, The University of Melbourne, Melbourne, VIC 3010, Australia; udaya@unimelb.edu.au; 3 Department of Computer Science and Software Engineering, School of Software and Electrical Engineering, Swinburne University of Technology, Melbourne, VIC 3122, Australia; pjayaraman@swin.edu.au

**Keywords:** IoT device integration, global discovery, semantic discovery, blockchain, autonomic integration, scalability, trusted, access control

## Abstract

The Internet of Things (IoT) incorporates billions of IoT devices (e.g., sensors, cameras, wearables, smart phones, as well as other internet-connected machines in homes, vehicles, and industrial plants), and the number of such connected IoT devices is currently growing rapidly. This paper proposes a novel Autonomic Global IoT Device Discovery and Integration Service (which we refer to as aGIDDI) that permits IoT applications to find IoT devices that are owned and managed by other parties in IoT (which we refer to as IoT device providers), integrate them, and pay for using their data observations. aGIDDI incorporates a suite of interacting sub-services supporting IoT device description, query, integration, payment (via a pay-as-you-go payment model), and access control that utilise a special-purpose blockchain to manage all information needed for IoT applications to find, pay and use the IoT devices they need. The paper describes aGIDDI’s novel protocol that allows any IoT application to discover and automatically integrate and pay for IoT devices and their data that are provided by other parties. The paper also presents aGIDDI’s architecture and proof-of-concept implementation, as well as an experimental evaluation of the performance and scalability of aGIDDI in variety of IoT device integration and payment scenarios.

## 1. Introduction

The Internet of Things (IoT) combines billions of IoT devices (e.g., sensors, RFIDs, cameras, wearables, smart phones, and other machine in industrial plants, homes, and vehicles) that sense the physical world and provide high value data observations (which we refer to as IoT data) that support the development of IoT applications. The number of connected IoT devices has grown from 7.74 billion in 2019 to 10.7 billion in 2021, and it is expected to reach about 25.44 billion by 2030 [1]. These IoT devices are owned by a variety of organizations or individuals who deploy them and utilize their IoT data for their own purposes. Currently, IoT provides no support for sharing IoT devices and their costs, and most IoT application procure, deploy, and maintain the sensor they need to collect the data they require. In this paper, we propose an Autonomic Global IoT Device Discovery and Integration (aGIDDI) service that permits IoT applications to discover, integrate, and use IoT devices owned and managed by any provider of IoT while sharing IoT device costs via a pay-as-you-go costing model. The paper makes the following novel contributions:

- Presents a pair of description and query sub-services that, respectively, allow IoT device providers to describe their IoT devices and their data observations and IoT applications to query the description of the available IoT devices so they can find the devices they need.
- Introduces sub-services permitting IoT applications interacting with aGIDDI to automatically integrate, pay for, and utilise IoT devices that are offered by one or more IoT device providers.
- Proposes a special-purpose blockchain, which we refer to as aGIDDI Blockchain, which is used by all aGIDDI sub-services to manage all the information required for discovering and integration of IoT devices, as well as for paying for their use. The use of a blockchain based in aGIDDI ensures that it is not owned or controlled by any IoT device provider, IoT application, IoT infrastructure provider or any other third-party. This is highly important in establishing the trust needed for all parties involved in global sensor sharing.
- Defines a novel protocol for IoT device discovery, integration, and payment, which we refer to as aGIDDI Protocol, that specifies all related interactions between (1) IoT device providers and IoT applications with aGIDDI, and (2) aGIDDI subservices with the aGIDDI Blockchain.
- Experimentally evaluates the performance and scalability of aGIDDI in integrating and controlling access to IoT devices under various IoT application workloads.

The rest of the paper is organised as follows: Section 2 discusses related work. Section 3 provides an overview of the aGIDDI service. The special-purpose aGIDDI Blockchain is described in Section 4, while Section 5 presents the sub-services of aGIDDI. Section 6 presents the aGIDDI protocol for IoT device discovery and integration. An implementation and an experimental evaluation of aGIDDI are presented in Section 7. Finally, Section 8 provides future research directions and the conclusion.

## 2. Related Work

To the best of our knowledge, we are not aware of any solution for autonomic and global discovering and integration of IoT devices. Existing solutions are siloed and proprietary (i.e., they are vendor-specific and are supported by cloud-based IoT platforms [2] and require application to deploy their own sensors) and lack automatic integration. Hydra middleware [3] allows IoT applications to integrate heterogenous IoT devices. Additionally, it uses semantic description to describe IoT devices and enable the discovery of IoT devices. In addition, Hydra middleware uses peer-to-peer (P2P) network technology to provide a trustworthy and secure service. However, the integration of IoT devices taking part is manual as it requires a developer to integrate them. Moreover, Hydra middleware has no incentive mechanism or ability to allow IoT device providers to get paid for sharing their IoT devices.

The Global Sensor Networks (GSN) middleware [4] provides a flexible IoT device discovery and integration. It supports a fast deployment of IoT devices and provides distributed querying of IoT data. GSN also offers more than forty integration wrappers for the most known IoT devices. Perera et al. [5] extended GSN with a plugin middleware to integrate IoT devices into IoT applications without the need for writing wrappers. OpenIoT [6] uses Semantic Sensor Network (SSN) [7] and GSN to enable IoT applications for performing semantic queries and integrating heterogenous IoT devices. To achieve this, OpenIoT proposed X-GSN, which is an improved version of GSN that supports integrating IoT devices that have been described via SSN [6]. However, neither OpenIoT nor GSN are providing an incentive mechanism to motivate IoT device providers to share their IoT devices. Furthermore, they do not support automatic integration for IoT devices, and they are not trusted.

Earlier trusted blockchains and other decentralized ledgers that have been developed specifically for IoT, e.g., [8] do not support the automatic and global discovery and integration of IoT devices. For example, IOTA [8] is a decentralized ledger designed specifically for providing a scalable mechanism to deal with the high flow of transactions of IoT. IoT Chain [9], which uses a private Ethereum Blockchain [10], provides a secured and authorized access to the registered IoT devices and can support basic IoT device discovery. Although both IOTA and IoT Chain are trusted, they do not support IoT device integration and do not provide an incentive mechanism to motivate IoT device providers to share their IoT devices. In addition, IOTA does not support access control for IoT devices.

Other research has focused on emerging Blockchain with IoT (e.g., [11]), but the main scope of this research is providing trust, privacy and security for IoT without providing discovery and integration of IoT devices. Dedeoglu et al. [12] proposed end-to-end trusted architecture for IoT based on blockchain. Their work focuses on providing trust on origin of IoT data, the communication among the IoT network, and the interaction between IoT applications and IoT devices. However, discovering and integration of IoT devices is not covered in this work. There is also no ability to pay for using other IoT devices. The authors of [11,13], proposed a lightweight scalable blockchain solution for IoT that provides privacy and security. However, semantic discovery and the integration of IoT devices are not addressed in this work. Moreover, this solution does not provide an incentive mechanism to motivate IoT device providers to share their IoT devices. Zhou, et al. [14] developed a Blockchain-based IoT system that is able to analyze IoT data in a decentralized environment. However, using blockchain nodes to analyze IoT data may impact on the scalability of the system. Additionally, IoT data may require to be stored in the blockchain, which impact the scalability of the system as well. In addition, semantic discovery and automatic integration of IoT devices are not covered in this paper. Furthermore, the paper does not provide an incentive mechanism to motivate IoT device providers to share their IoT devices.

Daza, et al. [15] developed CONNECT, which is contextual name discovery for blockchain-based services in IoT. CONNECT provides the ability to discover nearby IoT devices by sending a broadcast massage to all Blockchain nodes (note: IoT devices should be nodes in the blockchain) and wait for their return message. CONNECT has no generic measurement template or a description for IoT devices. CONNECT cannot semantically discover IoT devices or has a record of all IoT devices in the network, and it also has no automatic integration of IoT devices. Furthermore, CONNECT lacks an incentive mechanism to motivate IoT device providers to share their IoT devices.

Based on the above literature, non-blockchain and blockchain-based solutions lack automatic discovery and integration of IoT devices, as well as an incentive mechanism to motivate IoT device providers to share their IoT devices. Furthermore, non-blockchain approaches lack trust and the blockchain-based approaches store the IoT data in the blockchain, significantly impacting the performance of the blockchain. 

Our previous work presents Global IoT Device Discovery and Integration (GIDDI) Service [1,2,16] that addressed some of the related work limitations. GIDDI Service provides (1) specialized Blockchain to manage the semantic description of IoT devices and (2) decentralized marketplace (which is called GIDDI Marketplace) to provide the services of registering, manually querying, and paying IoT devices as well as provides necessary information to manually integrate IoT devices. GIDDI enables global sharing of IoT devices with different IoT applications. However, GIDDI falls short in providing the following: (1) automatic IoT device discovery as the IoT application developers must author and submit the SPARQL query needed for discovering the IoT devices that are appropriate for their applications, (2) automatic IoT device integration as application developments must provide the application code that uses the IoT device endpoints in their IoT device query results to integrate the IoT devices, and (3) payment transactions that ensure that IoT applications access IoT devices while their payments last. The aGIDDI service we propose in this paper provides fully automated interaction and payment transaction that addresses these limitations.

## 3. Autonomic Global IoT Device Discovery and Integration Service (aGIDDI)

**Figure 1:**
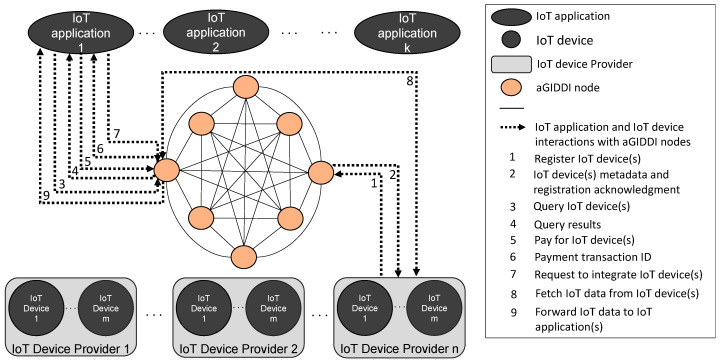
High-level architecture of aGIDDI.

As illustrated in Figure 1, the aGIDDI service is comprised of a collection of distributed nodes. aGIDDI nodes interact via the aGIDDI Blockchain that has been specifically designed to store and manage all information needed for IoT devices description, query, integration, and payment, which we collectively refer to as IoT device metadata.

**Figure 2:**
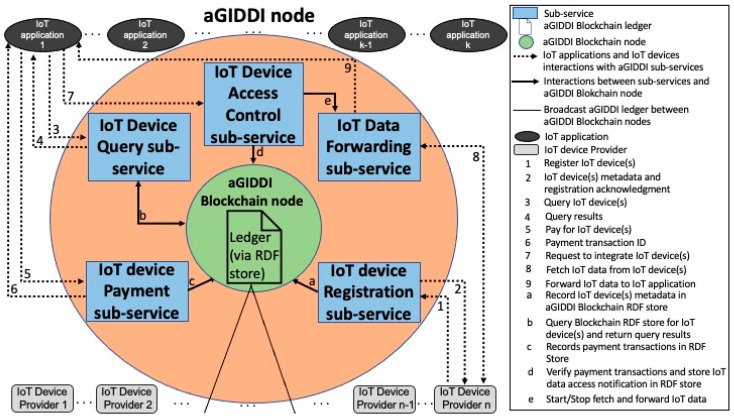
aGIDDI node architecture.

Figure 2 illustrates the architecture of the individual aGIDDI nodes in Figure 1. As shown in Figure 2, each aGIDDI node includes the following:

- An aGIDDI Blockchain node, which is responsible for blockchain-related functions including maintaining a ledger of all IoT device metadata, verifying generated blocks, and contributing to aGIDDI consensus. The aGIDDI blockchain is discussed further in Section 4.
- IoT device registration, IoT device query, IoT device payment, and IoT device access control sub-services, which allow IoT applications to query IoT devices are controlled and maintained by other parties (which we refer to as IoT device providers), integrate such IoT devices and their data observations, and pay for these. The IoT device data forwarding sub-service, which manages the flow of IoT device data from the IoT devices to the client IoT applications, is autonomically controlled by the IoT device access control sub-service. All aGIDDI sub-services use the aGIDDI Blockchain to manage and distribute the IoT device metadata (which, as we noted earlier, are used exclusively for IoT device description, query, integration, and payment) across all aGIDDI nodes. The aGIDDI sub-services are discussed further in Section 4.

IoT applications and IoT device providers can interact with aGIDDI via the sub-services in any of its nodes. For example, as illustrated in Figure 1, an IoT Device Provider may register its IoT devices via a specific aGIDDI node but forward IoT device data via another node. Similarly, an IoT application may query, pay, and integrate IoT devices via sub-services in different aGIDDI nodes.

IoT applications, IoT device providers, and the aGIDDI sub-services interact by using the aGIDDI protocol, which is described in detail in Section 6. To maintain compatibility with existing protocols that currently allow IoT applications to interact with IoT devices and obtain their data observations, aGIDDI (and more specifically, its IoT Data Fetching and Forwarding sub-service) supports many standard communications protocols that are commonly available in most existing IoT platforms. Without a loss of generality, in this paper, we assume that the IoT Data Forwarding sub-services utilises the standard MQTT protocol that is supported by virtually all existing IoT platforms. Other widely supported communication protocols are similarly supported, but they are outside of the scope of this paper.

## 4. aGIDDI Blockchain

This is a blockchain designed and implemented specifically for storing the IoT device metadata that are needed for IoT devices description, query, integration, and payment. In our earlier work [2,16], we presented how an earlier version of the aGIDDI blockchain (which is called GIDDI blockchain) is used to manage the semantic descriptions and IDs of IoT devices provided by other parties. In this paper, we explain how aGIDDI manages the metadata needed for its novel IoT device payment, integration, and access control functionality, which are being introduced by this paper. We also provide an overview of the functionality that has been incorporated into the aGIDDI from our earlier work [2,16] that enables IoT device description and query. In Section 4.1 and Section 4.2, we present the aGIDDI Blockchain nodes and aGIDDI Blockchain support for semantic description and query of IoT devices and their data, and the aGIDDI Blockchain support integrating and paying IoT devices.

### 4.1. aGIDDI Blockchain Nodes

The aGIDDI Blockchain is public and any node can join it the same way as adding a new node to Bitcoin [17] or many other similar public blockchains. Similarly, aGIDDI Blockchain nodes are responsible for generating new blocks, contributing to aGIDDI Blockchain consensus, and verifying newly generated blocks across the entire aGIDDI Blockchain.

Unlike other existing blockchains (e.g., Bitcoin [17]), aGIDDI Blockchain nodes provide the following novel features that support IoT device discovery, integration and payment:

- A copy of the aGIDDI Blockchain ledger that contains all IoT device metadata. This is a semantic ledger that records the SSN-based descriptions [2,16] of IoT devices and their data, as well as other metadata used for IoT device integration, payment, and access control.
- An RDF triple store that is used to store the above IoT device metadata records as triples organized in blocks, which we refer to as aGIDDI ledger blocks, or just aGIDDI blocks. aGIDDI’s RDF triple store provides highly efficient on-blockchain processing of semantic queries involving IoT device metadata.
- An interface that allows only the aGIDDI sub-services to access aGIDDI blocks in the aGIDDI blockchain.

When a new aGIDDI block is created in a node, this aGIDDI Blockchain node broadcasts newly generated aGIDDI block to all other aGIDDI Blockchain nodes to make the aGIDDI ledger consistent across all aGIDDI blockchain nodes. The broadcast mechanism is similar to other existing blockchains (e.g., Bitcoin [17]). IoT device description and query are discussed further next in Section 4.2.

### 4.2. aGIDDI Blockchain Support for IoT Device Description, Query, Integration and Payment

The aGIDDI Blockchain is specifically designed to support semantic IoT device query, integration, and payment. To support IoT device query, the IoT Device Providers record semantic descriptions of their IoT devices and their data in the aGIDDI ledger via the IoT Device Registration Sub-service (which is discussed further in Section 5.1). IoT device descriptions are based on the SSN [7] ontology that has been extended [2,16] to include IoT device provider ID, IoT device ID, sensor ID (an IoT device may incorporate several sensors), IoT device location, IoT device permission, IoT device payment required for using the IoT device, and endpoint for IoT device integration. As noted earlier in Section 4.1, aGIDDI nodes incorporate triple stores that store such IoT device metadata triplets in the local copy of the blocks that comprise the aGIDDI Blockchain ledger. This solution allows the IoT device query sub-service (which is discussed further in Section 5.1) to efficiently process SPARQL [18] IoT device-related queries that enable IoT applications to find the IoT devices they need.

**Figure 3:**
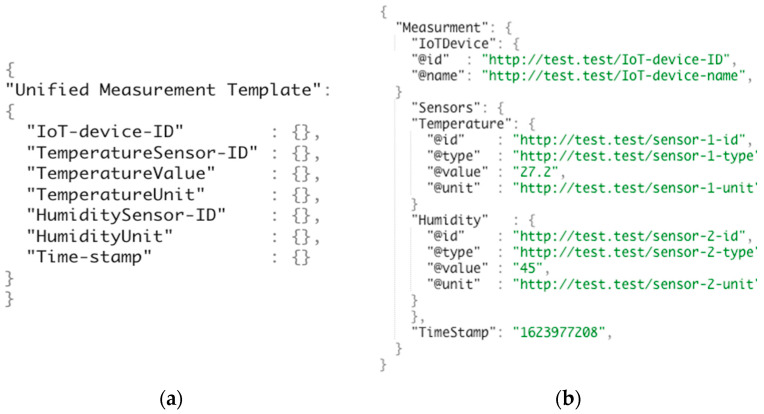
(**a**) Unified measurement template in IoT device metadata; (**b**) measurement of thermometer with two sensors example.

To allow IoT applications to automatically integrate and pay for the IoT devices they use, in this paper, we propose novel aGIDDI ontology extensions that include unified measurement templates, IoT device payment transactions, and IoT data access notifications that extend further the IoT device metadata we introduce in [2,16]. More specifically, a unified measurement template allows a IoT Device Provider to describe a specific IoT device and its data using concepts from the aGIDDI ontology. This unified measurement template is subsequently used by all IoT applications that use this IoT devices to integrate the IoT device and its data observations. All unified templates use concepts from/are consistent with the aGIDDI ontology. Figure 3a shows a sample unified measurement template that is provided by the provider of an IoT device. This template includes specific concepts from the aGIDDI ontology, i.e., the IoT device ID, IoT device name, the IDs of the IoT device’s temperature and humidity sensors, the types of the sensor observations from these sensors, the sensor observation units, as well as the observation timestamp. Figure 3b illustrates a sample observation from this IoT device that has been semantically annotated via the unified measurement template in Figure 3a and includes specific observation values for temperature and humidity and the corresponding timestamp value. Thus, the unified measurement template supports the autonomic integration of IoT devices.

**Figure 4:**
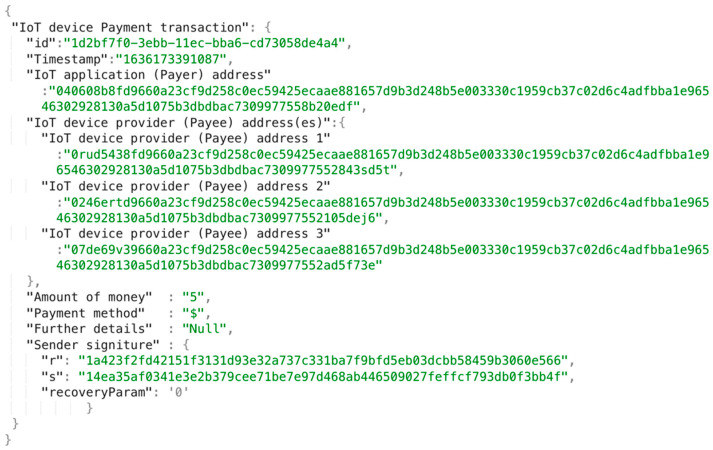
IoT device payment transaction with paying three IoT devices example.

To support IoT device payment, we propose adding a novel IoT device payment transaction concept in the aGIDDI ontology and recording payment transaction logs based on this concept in the aGIDDI ledger. These permit aGIDDI to manage IoT device payments via the aGIDDI Blockchain. Figure 4 shows a sample IoT device payment transaction that includes the following attributes: transaction ID, IoT application or payer ID (e.g., the payer’s public key, PayPal ID, bank account), IoT device provider ID or payee ID/(e.g., the payee’s public key, PayPal ID, bank account, etc.), amount of money paid, method of payment (e.g., Bitcoin payment, PayPal payment, bank transfer), transaction timestamp, and payer authorization/signature. The method of payment is provided by the payee/IoT device provider and is used by the IoT device payment sub-service (which is discussed further in Section 5.3) to choose the appropriate payment service. The transaction ID is generated by IoT device payment sub-service that is discussed further in Section 5.3. Other transaction attributes (e.g., additional payment methods, currencies, etc.) can be easily included. By including, the IoT device payment transaction in aGIDDI ontology, the aGIDDI Blockchain can maintain a log of IoT device payment transactions and use that to permit IoT applications to integrate selected IoT devices.

**Figure 5:**
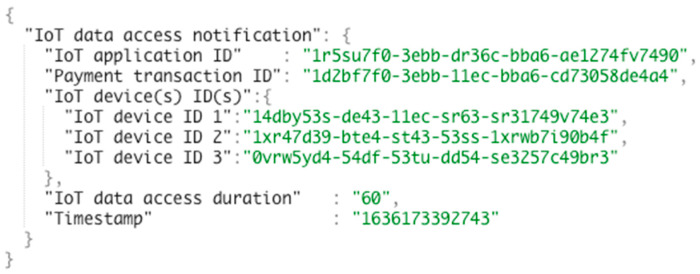
IoT data access notification with accessing three IoT devices example.

To maintain access to the IoT device data while the payment lasts, we have introduced the concept of IoT data access notification in the aGIDDI Ontology and aGIDDI records related access notification instances in the aGIDDI ledger. As illustrated in Figure 5, the IoT data access notifications include the following attributes: the IoT application ID, the payment transaction ID, the IDs of IoT device(s) the IoT application can access, the IoT data access duration or total number of data points, and the IoT timestamp of granting data access. The IoT application ID identifies the client IoT application. The payment transaction ID confirms a payment for accessing identified/listed IoT devices. The duration/total data point quota is used by IoT device access control sub-service (which is discussed further in Section 5.4) to control the access of the client IoT application to the listed IoT Device(s). The timestamp records the start of access to the data of the IoT devices listed.

To support IoT device query, integration, payment transactions and related data access, the GIDDI Blockchain blocks include public and encrypted sections. The public block section includes semantics description of the IoT devices and their data observations, IoT Device IDs, and Provider’s IDs. The encrypted block section includes the token/topic, and endpoint of the IoT devices. The public section is used for discovering (i.e., describing and querying) IoT devices, while the encrypted part is used for integrating, paying, and controlling access to IoT devices and their data. For example, the IoT device token/topic and endpoint are encrypted to prevent IoT applications from integrating and using IoT devices without a permission from aGIDDI (which is only granted after payment is received and while payment lasts). Only specific sub-services in the aGIDDI nodes can decrypt such encrypted information. The aGIDDI sub-services are presented next in Section 5.

## 5. aGIDDI Sub-Services

This section presents the aGIDDI sub-services that are the only components of the aGIDDI nodes that can access the aGIDDI Blockchain.

### 5.1. IoT Device Registration Sub-Service

This sub-service allows IoT device providers to register their IoT devices in aGIDDI in order to make them discoverable (via SPARQL queries) by IoT applications. As illustrated in Figure 2, the IoT Device Providers interact directly with the IoT device registration sub-service to provide their IoT device metadata and their information that is needed for payment transactions (as discussed in Section 4.2). Each provider can register multiple IoT devices together. Next, the IoT device registration sub-service generates the metadata for the registered IoT device(s), sends it to the IoT device provider, and submits it to aGIDDI Blockchain by inserting this in a block that needs to be verified by the aGIDDI Blockchain nodes. When the block verification is complete, the IoT device metadata are stored in the aGIDDI Blockchain ledger via the RDF triple store and can be queried via any aGIDDI node. To create the IoT device metadata, the IoT device registration sub-service (1) uses the aGIDDI Ontology (which was presented in Section 4.2 to generate publicly accessible unified measurement templates for all IoT devices being registered and (2) encrypt sensitive IoT device metadata, such as the IoT device token and end point. Once IoT device metadata of all IoT devices being registered are stored successfully in aGIDDI Blockchain, the IoT device registration sub-service sends an acknowledgment to the IoT device provider. The IoT device registration sub-service is also responsible for updating the IoT device description by creating a new IoT device metadata linking the IoT device ID of the earlier one. Note that in this case, both IoT device metadata versions will be visible on aGIDDI Blockchain as the information stored inside aGIDDI Blockchain is immutable (as in all other existing blockchains).

### 5.2. IoT Device Query Sub-Service

This sub-service supports querying the IoT device metadata that are stored in aGIDDI ledger by using one or more search conditions that are provided by each client’s IoT applications. Search conditions may involve IoT device attributes (e.g., the device type, location, cost, provider), the sensors that are incorporated in the IoT devices (e.g., sensor type, accuracy, range), and the data observations they produce (e.g., solar radiation, pressure, temperature). The IoT device query sub-service converts the submitted search conditions to a corresponding SPARQL query and processes this query efficiently using the built-in triple store in its aGIDDI node. For example, to find all IoT devices that have a temperature sensor with range greater than 100 degrees, an IoT applications submits the following search conditions as value name pairs that include concepts from the aGIDDI ontology: (Sensor) Type: Temperature, Range: >100. SPARQL query generation is based on the following principles:

- A SPARQL query contains two essential clauses, which are PREFIX and SELECT, and several elective clauses such as WHERE and FILTETR. The PREFIX clause defines the ontology that will be used in the query. By default, the PREFIX in our query-generating function is aGIDDI ontology.
- The SELECT clause is responsible for determining the structure of the query response. In this query example, the query structure consists of the “Type” and “Range” concepts.
- The WHERE clause is responsible for providing the graph pattern to match against the data graph, which is provided in the search conditions. The graph patterns in this query example are “Type” and “Range”. “Temperature” and “>100” are represented in the corresponding data graph.
- The FILTER clause contains Boolean expressions to filter the query results to match with the IoT application needs. In the query example, the filtration is applied on the range of temperature, which should be over 100 degrees.

**Figure 6:**
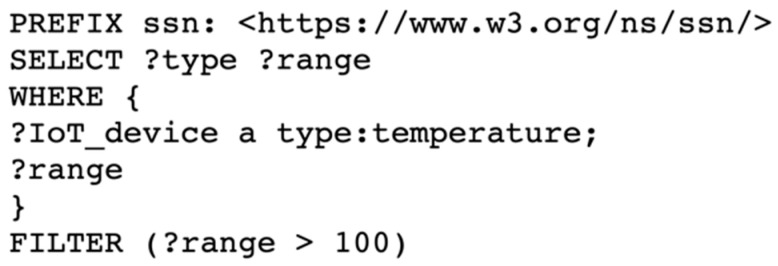
SPARQL query generated by IoT device query sub-service to support automatic discovery of IoT devices.

For this IoT device query example, the IoT device query sub-service generates the SPARQL query, as shown in Figure 6. When the query is executed, IoT device query sub-service returns a list of IoT devices that meet the IoT device search conditions and all public IoT device metadata that are available for each of these IoT devices.

The IoT device query sub-service also accepts direct SPARQL queries that are formulated using the aGIDDI ontology. While direct (i.e., non-generated by the query sub-service) SPARQL queries allow the use of the full expressive power of SPARQL that may be useful for some IoT applications, SPARQL queries are hard to formulate and may require expertise that may be beyond the skills of some IoT application developers [5].

### 5.3. IoT Device Payment Sub-Service

aGIDDI provides a novel pay-as-you-go mechanism that rewards IoT device providers with payments for sharing their IoT devices. This helps to increase the number of shared IoT devices that are available for IoT applications.

To enable IoT device payment, IoT device providers specify the IoT device cost per unit of time (e.g., per minute or P_minutes_) or unit of data (e.g., per Kbyte or P_kbyte_), as well as the method for receiving payment (e.g., PayPal, Bitcoin, bank transfer), when they register their IoT devices in aGIDDI via the IoT device registration sub-service, which was discussed in Section 5.1.

IoT applications use the IoT device payment sub-service to pay for the IoT devices they select from the query results they obtain from the IoT device query sub-service, which was presented in Section 5.2. The IoT device payment sub-service computes the total payment P_total_ for using IoT devices by considering: (1) the cost of using IoT devices per minute P_minutes_, multiplied by the number of minutes N_minutes_, and (2) the cost of IoT data per Kbyte P_kbyte_ multiplied by the number of delivered Kbytes N_kbytes_, as represented by:

P_total_ = P_kbyte_ × N_kbytes_ + P_minute_ × N_minutes_. (1)

As soon as the IoT application/payer makes the IoT device payment, the IoT device payment sub-service verifies the payment and creates an IoT device payment transaction, as discussed in Section 4.2. To perform the latter, the IoT device payment sub-service first generates an IoT device payment transaction ID and a transaction timestamp. Next, the IoT device payment sub-service includes these and the IoT device(s) and provider details in an IoT device payment transaction, stores the transaction in an aGIDDI block, and submits this to the aGIDDI Blockchain. Each IoT device payment transaction can include payment for multiple IoT devices that are shared by the same provider. The IoT device payment transactions are then used by IoT device access control sub-service (which is discussed further in Section 5.4) to ensure the payment and to give permission to the payer IoT application to access the IoT devices it has paid for.

If an IoT application has not paid for an IoT device, the IoT device access control sub-service rejects IoT device integration requests (i.e., withholds the IoT device endpoint) from the application. The IoT application can only gain access to the IoT device if it uses the IoT device payment sub-service to pay for the IoT device and the payment sub-service has created a transaction that has been successfully verified and added to the aGIDDI Blockchain ledger. This creates an incentive mechanism for IoT device providers that motivates them to share more IoT devices.

### 5.4. IoT Device Access Control Sub-Service

IoT applications gain access to the IoT device(s) they have paid for by sending an integration request to the IoT device access control sub-service. Integration requests include the following: the ID of the requesting IoT application, the IDs of the target IoT device(s), the ID of the IoT device payment transaction that recorded the payment for these IoT device(s), and the metadata of targeted IoT devices that are recorded in the aGIDDI Blockchain ledger. The IoT device access control sub-service verifies the IoT device payment transaction by matching the target IoT device IDs with the device IDs recorded in the IoT device payment transaction in the aGIDDI Blockchain ledger. If all target IoT device IDs match the IDs in a recorded payment transaction, the payment is verified. The IoT device access control sub-service can grant the access of IoT device(s) to the IoT application for either a duration of time (i.e., if the IoT application paid for accessing IoT device(s) per unit of time) or data size (i.e., if the IoT application paid for accessing IoT device(s) per unit of data size) based on the information in the payment transaction log, which contains the recorded payment transactions (i.e., part of IoT device metadata) inside aGIDDI Blockchain ledger as explained in Section 4.2. To illustrate, IoT application A requests for accessing IoT device(s) D and paid for them by payment transaction PT. IoT device access control sub-service verifies PT by checking that PT has recorded in the payment transaction log PL and D’s ID match the ID(s) recorded in PL, as explained earlier. Once the payment is confirmed, there are two scenarios that the IoT device access control sub-service can follow:

- If A paid for accessing D per unit of time, the IoT device access control calculates the duration of time T of accessing D. Then, each time D pushes new IoT data, the IoT device access control checks if the current_time − timestamp_in_PT ≤ T is true; if so, it allows the IoT data flow from D to A. Otherwise, it stops the IoT data flow.
- If A paid for accessing D per unit of data size, the IoT device access control calculates the total IoT data size S that should flow from D to A. Then, it maintains IoT data counter C, which initially starts with zero (C = 0) and then increments with the size of IoT data (C = C + size of new IoT data) every time D pushes new IoT data. The IoT device access control checks if C ≤ S is true; if so, it allows the IoT data flow from D to A. Otherwise, it stops IoT data flow.

The IoT device access control sub-service calculates T and S by dividing the amount of money paid by A from PT over the cost of accessing D per unit of time or data size from IoT device metadata as can be seen from the following formulas:

T = Amount of Money paid/Cost per unit of time(2)

S = Amount of Money paid/Cost per unit of data size(3)

For example, if the cost of accessing D was 10 cents per hour and A paid 50 cents, that means T = 5 h. Additionally, if the cost of accessing D was 2 cents per MB and paid 50 cents, that means S = 25 MB.

The IoT device access control sub-service allows IoT data flow from D to A by instructing the IoT data-fetching and -forwarding sub-service (Section 5.5) to fetch IoT data from D and forward it to A. It also stops the IoT data flow by instructing the IoT data-fetching and -forwarding sub-service to stop fetching IoT data from D and forward it to A. Finally, the IoT device access control submits IoT data access notification to aGIDDI Blockchain to record the information of integrating A to D in aGIDDI blockchain ledger.

### 5.5. IoT Data Fetching and Forwarding Sub-Service

The IoT data-fetching and -forwarding sub-service is responsible for controlling the flow of IoT data from each specific IoT device to its client IoT applications. When the IoT device access control sub-service verifies the payment of IoT application A for accessing the IoT device(s) D, it instructs the IoT data-fetching and -forwarding sub-service used in the encrypted endpoint and other standard IoT device communication protocol specific information, such as the Topic for MQTT, so D can access D’s data via this protocol, and then encapsulates the received IoT data with unified measurement template retrieved from D’s metadata. Next, it forwards the encapsulated measurement data to A for the period the payment last made by A. It continues the IoT data flow from D to A until it receives stop instruction from IoT device access control sub-service as explained in Section 5.4.

## 6. aGIDDI Protocol

aGIDDI protocol allows IoT applications to discover, pay and integrate variety of heterogeneous IoT devices, and may use heterogeneous configuration/communications protocols from virtually any vendor. To achieve this, aGIDDI relies on the array of standard protocols provided by widely used IoT platforms (e.g., Azure IoT, Cumulocity, MindSphere, etc.), which are currently handling naming, communication, and authentication of heterogeneous multi-vendor devices well enough to support the great majority of available IoT devices. To illustrate this, in this paper, we focus on IoT device naming and communication via the MQTT messaging protocol that is currently supported by virtually all available IoT platforms and the majority of available IoT devices. While the aGIDDI protocol we present in the following steps is MQTT specific (specifically, Step 5 relies on MQTT), the aGIDDI can be similarly extended to utilise additional alternative protocols (e.g., CoAP). However, extending the implementation of the aGIDDI protocol is outside the main focus of this paper, which is on aGIDDI autonomic integration that can be sufficiently illustrated via MQTT. Please note that many widely used IoT platforms (e.g., Cumulocity) (i.e., provide implantation of MQTT) offer IoT device data encryption and authentication, while others (e.g., MindSphere) offer specialized devices (e.g., MindConnect devices) that provide related hardware-enabled support for these.

**Figure 7:**
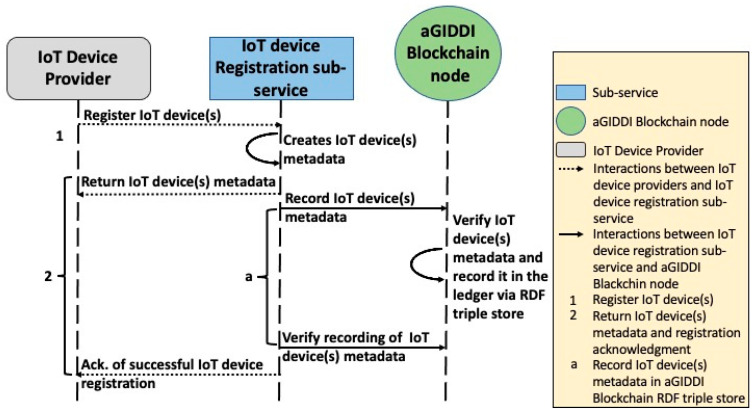
aGIDDI protocol for IoT device registration.

**Figure 8:**
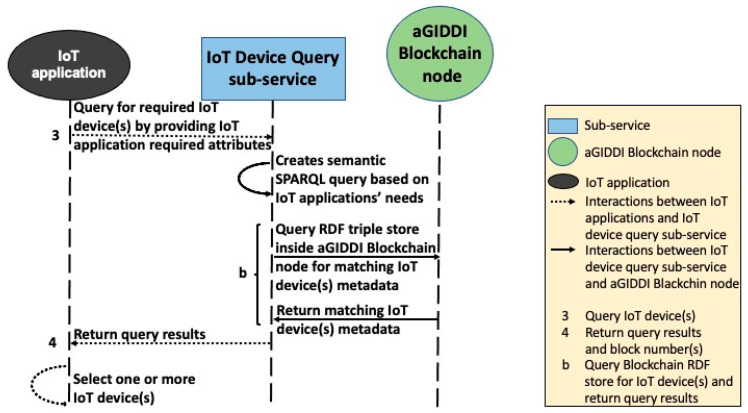
aGIDDI protocol for IoT device query.

**Figure 9:**
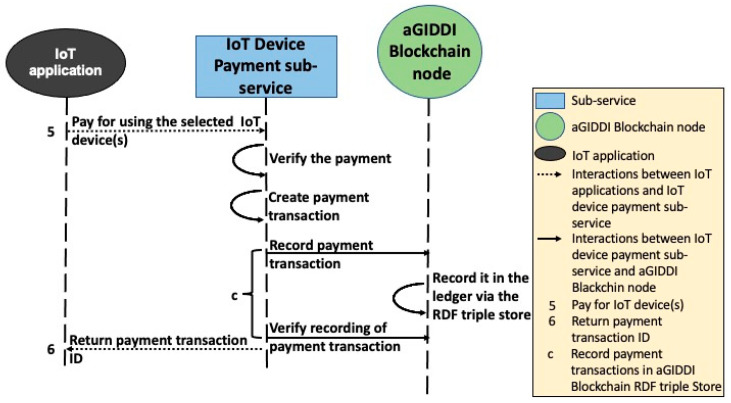
aGIDDI protocol for IoT device payment.

**Figure 10:**
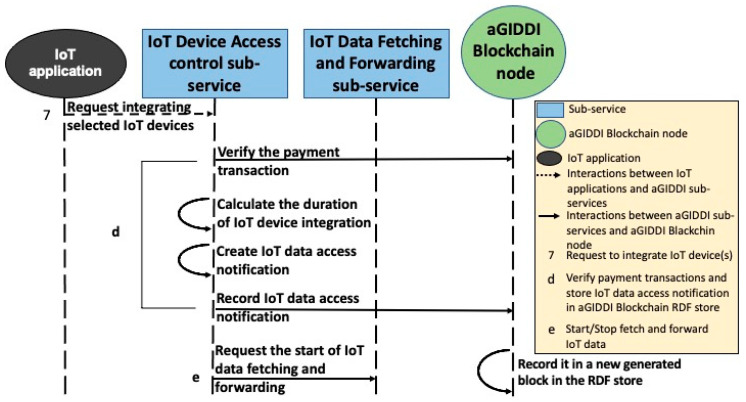
aGIDDI protocol for IoT device integration (controlling the access of IoT devices).

In this paper, we propose that (1) IoT applications use IoT platforms of their choice for IoT device data storage and analysis, (2) IoT applications use aGIDDI protocol to discover, integrate, and pay for IoT devices and their data, and (3) aGIDDI protocol uses the IoT platform’s MQTT protocol to initiate and control the IoT data flow from the IoT devices. The following steps along with Figure 7, Figure 8, Figure 9 and Figure 10 explain the aGIDDI protocol from registering IoT devices by IoT device providers and ending by receiving measurements by IoT applications:

**Step 1:** IoT device providers use the IoT device registration sub-service endpoint to register their IoT devices. The IoT device registration sub-service expects a POST request and a JSON body that includes the IoT device information (e.g., type, name, and measurement unit) and IoT device provider PK which is represented by interaction 1 in Figure 7. Once the POST request is successfully submitted, the IoT device registration sub-service creates semantic IoT device metadata based on aGIDDI ontology filled with IoT device semantic description, IoT device identities, and IoT device provider PK. Then, the IoT device registration sub-service returns the IoT device metadata to the IoT device provider and submits it to aGIDDI Blockchain node to be recorded in aGIDDI ledger via RDF triple store. Once the aGIDDI Blockchain approves the IoT device metadata and records it in an aGIDDI block inside aGIDDI ledger, the IoT device becomes visible for IoT applications as represented by interaction 2 and a in Figure 7. Afterward, the IoT device registration sub-service checks if the IoT device metadata is successfully recorded by searching for it inside aGIDDI ledger via RDF triple store consistently every few seconds. Once the IoT device metadata are recorded successfully, the IoT device registration sub-service returns an acknowledgment of successful IoT device registration to the IoT device provider (interaction 2 in Figure 7).

**Step 2:** IoT applications use the IoT device query sub-service to find the required IoT devices. The IoT device query sub-service expects POST request and JSON body including value name pairs for the needed attributes as represented by interaction 3 in Figure 8. The IoT device query sub-service creates a semantic query for finding the best match IoT devices based on IoT application needs. The IoT device query sub-service queries aGIDDI ledger inside the aGIDDI Blockchain node via RDF triple store semantically and obtains query results back, as we can see in interaction b in Figure 8. Then, it returns a list of IoT device metadata to the IoT application in order to select one or more IoT devices for integrating them (see interaction 4 from Figure 8).

**Step 3:** IoT applications interact with the IoT device payment sub-service to pay for using the required IoT devices. The IoT device payment sub-service expects a POST request and JSON body including IoT application (payer) address, IoT device provider(s) (payee(s)) address(es), amount of money, the method of payment, payer signature/authorization, and other details may be required by different payment methods, as represented by interaction 5 in Figure 9. The sub-service allows IoT applications to pay by using different methods such as Bitcoin, PayPal, or credit card. The IoT device payment sub-service asks the IoT applications to authorize their payments by providing the card details, PayPal credentials, or digital signature. Then, the sub-service verifies the payment by contacting the authority that is responsible for the payment. Next, the IoT device payment sub-service creates ID and timestamp for the IoT device payment transaction and submits it to aGIDDI Blockchain node to be stored in aGIDDI ledger via RDF triple store. Once the payment transaction is stored successfully in aGIDDI Blockchain, the IoT device payment sub-service sends the IoT device payment transaction ID to the IoT application (see interaction 6 from Figure 9) to be used later for obtaining the IoT device integration permit from the IoT device access control sub-service.

**Figure 11:**
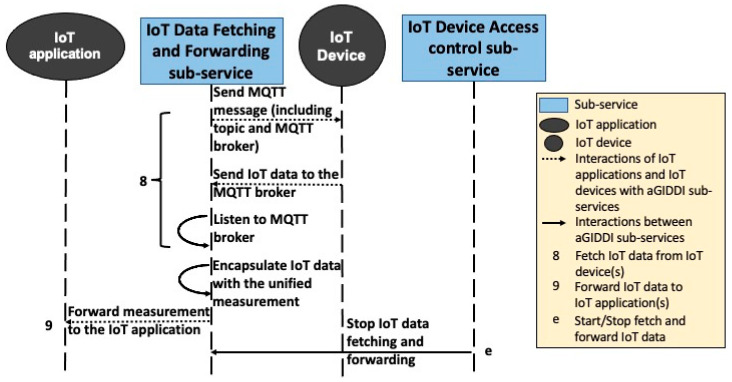
aGIDDI protocol for IoT device integration (fetching IoT data from IoT devices and forwarding it to IoT application).

**Step 4:** IoT applications interact with the IoT device access control sub-service to gain permission for integrating the required IoT devices. The sub-service expects a POST request along with JSON body that includes the required IoT device IDs, IoT application ID, payment transaction ID, and all related IoT device metadata, as represented by interaction 7 from Figure 10. The IoT device access control sub-service verifies the IoT device payment transaction, as explained in Section 5.4. Once the checking is passed, it calculates the duration of IoT data access or the total size of IoT data based on the cost concept from each IoT devices metadata and the amount of payment for each IoT device from the IoT device payment transaction. Then, it creates IoT data access notification based on aGIDDI Ontology and submits it to aGIDDI Blockchain node in order to store it in aGIDDI ledger via RDF triple store. Next, it sends a request to the IoT data fetching and forwarding sub-service to start, as we can see from Figure 10, interaction d. Once the duration of IoT data access expires or the total IoT data size reaches, the IoT device access control sub-service sends a request to stop fetching and forwarding IoT data (see Figure 11, interaction e).

**Step 5:** The IoT data-fetching and -forwarding sub-service sends MQTT massage to the selected IoT devices to inform them to publish their IoT data to a specific MQTT broker and temporary topic, as represented by interaction 8 in Figure 11. The MQTT broker is selected by the IoT data-fetching and -forwarding sub-service, the token is randomly and temporarily generated for this specific integration, and the duration is managed by the IoT device access control sub-service. IoT devices start sending IoT data to MQTT broker. The IoT data-fetching and -forwarding sub-service listens to the MQTT broker to receive the IoT data and then encapsulates it with the unified measurement template by using information from IoT device metadata. Afterwards, it starts forwarding the measurement to the IoT application, as we can see from Figure 11, interaction 9. The IoT device metadata and the measurement are written semantically, so IoT applications can understand the IoT data and convert it into the preferred unit or standard as well as converting the measurement into the preferred measurement template. Finally, the IoT device access control sub-service sends a request to the IoT data fetching and forwarding in order to end the integration once the duration time expires (see Figure 11, interaction e). The IoT data-fetching and -forwarding sub-service monitors the integration process in case one or more IoT devices loses connection for any reason to later decide a compensation between IoT device providers and the IoT application in our future work.

## 7. Implementation and Experimental Evaluation of aGIDDI

This section presents an implementation along with large-scale experiments to evaluate the performance and scalability of aGIDDI to integrate a large number of IoT devices with a large number of IoT applications.

### 7.1. Proof of Concept Implementation of aGIDDI

We have used NodeJS to implement aGIDDI Blockchain and all aGIDDI sub-services including IoT device registration, IoT device query, IoT device payment, IoT device access control, and IoT data fetching and forwarding. The Universal Unique Identifier (UUID) is implemented to provide a unique identification for IoT devices, aGIDDI ledger Blocks, IoT device payment transactions, IoT device metadata, and IoT data access notifications. SHA-256 hash function provides hash values to chain the blocks in aGIDDI Blockchain ledger. The elliptic cryptography service [19] generates unique public and private keys for each IoT device provider, aGIDDI node, and IoT application in order to uniquely identify them and verify their digital signature. The peer-to-peer communication inside aGIDDI Blockchain is supported by a Web socket protocol as it provides bidirectional communications between aGIDDI nodes. N3 RDF triple store [20] was implemented in each aGIDDI Blockchain node to store IoT device metadata triples in aGIDDI Blockchain ledger. Only for testing the functionality of aGIDDI Blockchain, Proof of Work (PoW) consensus algorithm [17] is applied to controls the time of generating new aGIDDI blocks. PoW is being used successfully by several leading blockchains [21], including Bitcoin [17] and Ethereum 1.0 [10]. PoW is easy to implement and provides the required security for aGIDDI Blockchain. The IoT device metadata developed by using Protégé software [22] is based on Semantic Sensor Network (SSN).

We employed the Comunica SPARQL query engine [23] to implement the IoT device query sub-service. Hyper Text Transfer Protocol (HTTP) enabled the communication with (1) IoT applications to query IoT devices and request integrating IoT devices, and (2) IoT device providers to register their IoT devices. MQTT protocol was used for communicating between the IoT data fetching and forwarding sub-service and IoT devices as well as between the IoT data-fetching and -forwarding sub-service and IoT applications. MQTT.js library was used to implement the MQTT protocol in each aGIDDI node. Table 1 includes implementation details for all subservices/components of aGIDDI.

#### 7.1.1. Implementation of Simulated IoT Devices

We have implemented a large number of simulated IoT devices in order to use them for evaluating the scalability of aGIDDI. The simulated IoT devices were used as it is hard to use a large number of real IoT devices in our evaluation. Each simulated IoT device can be represented by a piece of code that generates IoT data in a fixed duty-cycle such as real IoT devices. IoT-Data-Simulator [24] has been used to create the simulated IoT devices.

#### 7.1.2. Implementation of Real IoT Devices

To complement the simulated IoT devices, we have implemented a number of real IoT devices for our evaluation. Although the number of real IoT devices is small compared to the number of simulated IoT devices, the implementation of these real IoT devices can help to measure the impact of the limited resources (e.g., CPU and memory) of IoT devices on our evaluation. For this purpose, we have implemented five Arduino UNO (i.e., using ATmega328P with 32 Kbyte of flash memory) with temperature sensor DHT22 and programmed them to generate IoT data with the same amount and duty-cycle of the simulated IoT devices.

### 7.2. Evaluation of aGIDDI

In this section, we present the results of large-scale experimental evaluations conducted to evaluate the performance and scalability of aGIDDI. In our previous work [2], we evaluated the performance and scalability of the semantic discovery (registration and query) of IoT devices. In this paper, evaluate the performance and scalability of aGIDDI to integrate IoT devices automatically.

#### 7.2.1. Experimental Setup

Nectar research cloud, which is Australian’s national research cloud that provides cloud computing for Australian researchers is used to run aGIDDI for these experiments. Twenty aGIDDI nodes (includes aGIDDI Blockchain nodes and aGIDDI sub-services) were deployed to run and manage aGIDDI. Each aGIDDI node used the NeCTAR Ubuntu 16.04 LTS (Xenial) amd64 [v37] operating system. Additionally, each aGIDDI node has a public IP, 16 GB of RAM, and 1 TB of Hard Disk. A Windows-based computer and Gatling load test Software [25] were used to generate the necessary IoT devices integration requests and IoT device payment transaction (i.e., uses IoT device access control, IoT data fetching and forwarding, IoT device payment sub-services). The Windows-based computer has an 8 Core i7-7700 CPU @3.60 GHz and 16 GB of RAM.

#### 7.2.2. Assumptions of the Experimental Evaluation

In our evaluation of aGIDDI (Section 7.2.3 and Section 7.2.4), we have made the following simplifying assumptions that do not significantly impact the overall evaluation outcomes:

- All IoT devices use the same MQTT broker.
- All IoT devices have the same fixed duty-cycle.
- Measurements include Steps 4 and 5 of the aGIDDI protocol in Section 6 (i.e., the total response time of the IoT device access control sub-service and IoT data fetching and forwarding sub-service needed for integrating each IoT device), but do not include the overhead in Steps 1, 2 and 3 (i.e., the IoT device registration, query, and payment overhead) that take place earlier. As noted earlier, an evaluation of steps 1 and 2 (i.e., query and registration) is presented in [2], while a Step 3 evaluation is in progress and will be included in a follow-up publication.

#### 7.2.3. Experiments Conducted to Evaluate aGIDDI

In the following experiments, we measure the response time of integrating IoT devices (Step 4 and 5 in Section 6) using simulated and real IoT devices. The metrics computed during the experiments include the maximum response time and the mean response time of integrating IoT devices in different scenarios. More specifically, in Section 7.2.3.1, we vary the number of simulated IoT devices, while in Section 7.2.3.2, we vary the number of IoT applications with simulated IoT devices. In these two experiments, we have used a large number of simulated IoT devices and large number of IoT applications to test the scalability of aGIDDI. Due to the limitation of the public MQTT broker (HiveMQ public MQTT broker [26] was used in these experiments), the maximum number of integrated IoT devices was 200,000. We faced a broker failure when we tried to integrate more IoT devices. Finally, in Section 7.2.3.3, we used real IoT devices to study the impact of their limited resources on the aGIDDI protocol.

##### 7.2.3.1. Measuring the Impact of Varying the Number of Simulated IoT Devices

**Figure 12:**
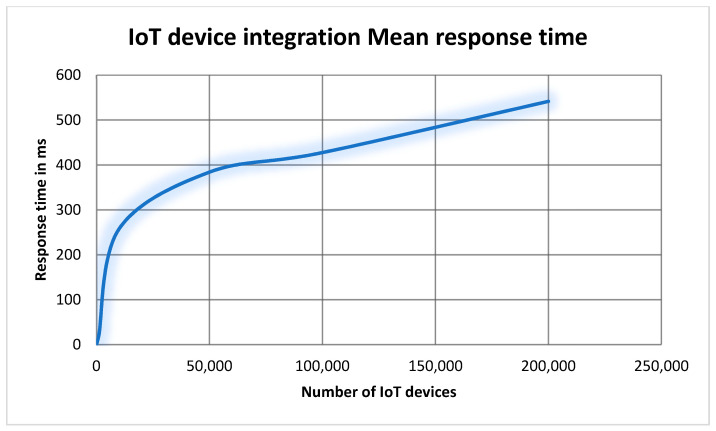
The relationship between the number of integrated IoT devices and the mean response time.

**Figure 13:**
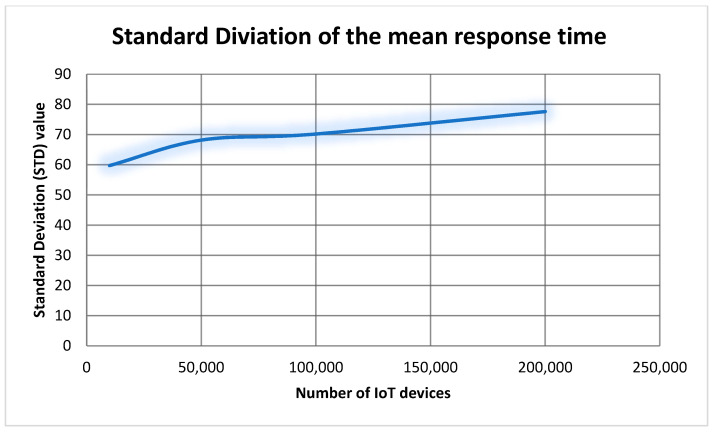
The relationship between the number of integrated IoT devices and the standard deviation.

This experiment conducted several tests to evaluate the mean response time and maximum response time of integrating simulated IoT devices. The results of the six test outcomes are presented in Table 2. As we can see from Table 2 and Figure 12, the mean response time increased gradually with respect to the increasing of IoT devices. Additionally, we did not face a breaking point when the number of IoT devices increased. That means aGIDDI is capable to handle large number of IoT devices without majorly impacting the mean time response. Although the mean response time of integrating IoT devices shows acceptable results, we need a statistical method to evaluate the response time of integrating IoT devices. Therefore, we use standard deviation (SD) to measure the amount of variation of the response time. From Table 2 and Figure 12 and Figure 13, we can clearly see that the relation between the mean response time and its SD is linear with respect to increasing the number of IoT devices. At 100 IoT devices, the mean response time is 2ms and the SD is 1.82, which means 68% of IoT device integration requests were processed in a range of mean response time ± SD (i.e., 0.18 ms to 3.82 ms). Additionally, at 200,000 IoT devices, the mean response time is 541.5 ms and the SD is 77.59. That means 68% of IoT device integration requests were processed in a range of 0.46 s and 0.61 s. That means the variation of the mean response time is low and it is not likely to be impacted by increasing the number of integrated IoT devices. Furthermore, the SD numbers from Table 2 indicate that the maximum response time happened for less than 1% of the total IoT device integration requests because 99% of the integration requests through the experiment were from a range of mean response time ± 3SD. Finally, we can indicate from Table 2 that the size of aGIDDI Blockchain is increasing with respect to the number of IoT devices registered (i.e., IoT device metadata stored in aGIDDI Blockchain).

##### 7.2.3.2. Measuring the Impact of Varying the Number of IoT Applications with Simulated IoT Devices

In this experiment, we conducted several tests to evaluate the mean response time when we increase the number of IoT applications. We started with one IoT application until 1000 IoT applications. We have noticed that the aGIDDI mean response is increased with respect to the increased number of IoT applications. However, increasing the number of IoT applications leads to an increase in the total integrated simulated IoT devices. To illustrate this, if we have 10 IoT applications trying to integrate 10 IoT devices each, this leads to the total integrated IoT devices being 100. Therefore, we compared the response time of the total integrated IoT devices with respect to the increasing number of IoT applications (see Table 3: test (2 and 6), (3 and 7), and (4 and 8)); we noticed that increasing the number of IoT applications has no major impact on the aGIDDI mean response time, because the total integrated IoT devices is same. That means one IoT application integrates 100 IoT devices is similar to 10 IoT applications integrate 10 IoT devices each. Therefore, aGIDDI has the ability to handle a large number of IoT applications, the same as the ability to integrate a large number of IoT devices. The maximum number of IoT applications we could reach was 1000 IoT applications to integrate 2000 IoT devices each (i.e., total 200,000 integrated IoT devices) due to the limitation of the public MQTT broker that we used for this experiment (as we mentioned above in Section 7.2.3). From Table 3, it is clear to see that the size of the aGIDDI Blockchain is not impacted by the variation in the number of IoT applications, but it increased by increasing the number of IoT devices. This is expected as IoT applications have no information/description stored in aGIDDI Blockchain, while each registered IoT device should have metadata stored in aGIDDI Blockchain.

##### 7.2.3.3. Measuring the Impact of Resource Limitations in Real IoT Devices

In this experiment, we have used five real IoT devices to evaluate the ability of aGIDDI in terms of integrating real IoT devices. Real IoT devices have limited resources (e.g., CPU and memory) that need to be considered in our evaluation. Therefore, we evaluated aGIDDI to integrate five IoT devices (i.e., Arduino UNO with temperature sensor DHT22), which are programmed to generate IoT data with the same amount and duty-cycle of the simulated IoT devices that used in previous experiments. As we can see from Table 4, the mean response time of integrating these five IoT devices was about 1 ms with a maximum response time of 5 ms. Additionally, integrating five IoT devices by one IoT application is almost the same as five IoT applications integrating one IoT device each, as we can see from test 1 and 2 from Table 4.

#### 7.2.4. Experimental Results and Analysis

The results from the first experiment show that the IoT device integration mean response time has a linear relationship with the increasing number of IoT devices. Additionally, the variation of IoT device integration mean response time is low compared to its value. Furthermore, the maximum response time recorded only happened for less that 1% of the total IoT device integration requests. The results from the second experiment show that there is a linear relationship between the mean response time of integrating IoT devices and the number of IoT applications. In fact, increasing the number of IoT applications has no effective impact on the mean response time of integrating IoT devices, but it results in increasing the number of integrated IoT devices, which leads to an impact on the mean response time.

We observed that the maximum response time of integrating IoT devices happens when many IoT device integrations are processed at the same time, which reduces the performance of IoT device access control sub-service and IoT data-fetching and -forwarding sub-service.

The results from real IoT devices show that the limited resources (e.g., CPU and Memory) of real IoT devices such as Arduino UNO have no major impact on the scalability of aGIDDI protocol. That means we can predict that the results from evaluating the integration of a large number of simulated IoT devices can be obtained from the same number of real IoT devices.

In this evaluation, we did not add a comparison between our novel trusted blockchain-based autonomic IoT device discovery and integration (aGIDDI) with other existing blockchain-based solutions for IoT because they are not allowing IoT applications to semantically discover IoT devices and integrate them autonomically.

Based on this evaluation, we can say that (1) aGIDDI has reliable performances to integrate a large number of IoT devices and IoT applications, and (2) aGIDDI is a scalable service for autonomic IoT device integration.

## 8. Conclusions and Future Work

In this paper, we proposed a novel autonomic discovery and integration service for IoT devices that significantly extends our previous work Global IoT Device and Integration (GIDDI) Service [2,16] to achieve Autonomic and Global IoT Device Discovery and Integration (aGIDDI) Service. aGIDDI consists of aGIDDI Blockchain and several sub-services which are IoT device registration, IoT device query, IoT device payment, IoT device access control, and IoT data fetching and forwarding. aGIDDI allows IoT applications to discover, integrate, pay, and use IoT devices for their own purposes autonomically and globally. Additionally, aGIDDI controls the access of IoT devices and prevent IoT applications to integrate IoT devices without paying them (unless IoT devices are provided for free). In addition, aGIDDI provides an incentive-based mechanism that ensures revenue for IoT device providers for sharing their IoT devices with IoT applications. Furthermore, aGIDDI uses semantic IoT device discovery and integration protocol called aGIDDI protocol that enables discovering and integrating any type of IoT devices with IoT applications that use any IoT platform autonomically. Furthermore, we inducted large-scale experiments to evaluate the performance and scalability of aGIDDI to integrate a large number of IoT devices. Based on the experimental evaluation, we believe that aGIDDI provides scalable autonomic IoT device integration. Future research directions include (1) extending aGIDDI to provide build-in IoT device authentication instead of exclusive relying on IoT platforms, and (2) studying the impact of aGIDDI’s power consumption. Further research in this field involves utilizing aGIDDI in industry 4.0, which involves complex IoT devices; smart cities, which contain mobile IoT devices; and industrial applications (e.g., monitoring and supply chains), which contain a large number of different IoT devices.

## Figures and Tables

**Table 1:** aGIDDI implementation details.

Sub-Services/Components	Language/Software	Technology	Algorithm/Protocol
aGIDDI Service	NodeJS	UUID, SHA-256, Elliptic cryptography, Timestamp	Web socket, HTTP, Proof of Work
IoT device registration	NodeJS	UUID, Timestamp	HTTP
IoT device query	NodeJS	Comunica SPARQL	HTTP
IoT device payment	NodeJS	Timestamp, digital signature	HTTP
IoT device access control	NodeJS	Timestamp	HTTP
IoT data fetching and forwarding	NodeJS	MQTT.js, Timestamp	MQTT
IoT device metadata	Protégé, JSON-LD	SSN, aGIDDI Ontology	-
RDF triple store	npm package	Semantic	N3

**Table 2:** This table shows the results of each test in the first experiment.

Test Number	No. of IoT Devices	Mean Response Time	Max Response Time	Standard Deviation	aGIDDI Blockchain Size
1	100	2 ms	31.6 ms	1.82	2.38 MB
2	1000	19.5 ms	204.6 ms	3.2	23.92 MB
3	10,000	257.6 ms	61,539 ms	59.71	239.11 MB
4	50,000	384 ms	61,645.6 ms	68.15	1.164 GB
5	100,000	427.5 ms	62,879.5 ms	70.17	2.401 GB
6	200,000	541.5 ms	66,508.5 ms	77.59	4.802 GB

**Table 3:** Results of each test in the second experiment.

Test Number	No. of IoT Applications	No. of IoT Devices	Total Number of IoT Devices	Mean Response Time	Max Response Time	aGIDDI Blockchain Size
1	1	100	100	2 ms	31.6 ms	2.38 MB
2	1	10,000	10,000	257.6 ms	61,539 ms	239.11 MB
3	1	100,000	100,000	427.5 ms	62,879.5 ms	2.401 GB
4	1	200,000	200,000	541.5 ms	66,508.5 ms	4.802 GB
5	10	10	100	3 ms	33.8 ms	0.23 MB
6	100	100	10,000	275 ms	61,924.2 ms	2.38 MB
7	1000	100	100,000	435 ms	62,721.4 ms	2.38 MB
8	1000	200	200,000	545 ms	66,752.9 ms	4.79 MB

**Table 4:** Results of each test in the real IoT devices experiment.

Test Number	No. of IoT Applications	No. of Real IoT Devices	Total Number of Real IoT Devices	Mean Response Time	Max Response Time
1	1	5	5	1 ms	5 ms
2	5	1	5	1 ms	5.1 ms

## Data Availability

Not applicable.
